# Pre-operative serum vascular endothelial growth factor can select patients for adjuvant treatment after curative resection in colorectal cancer

**DOI:** 10.1054/bjoc.2000.1508

**Published:** 2000-11-22

**Authors:** K-F Chin, J Greenman, E Gardiner, H Kumar, K Topping, J Monson

**Affiliations:** Academic Surgical Unit and Department of Mathematics, Department of Mathematics, The University of Hull, Hull, UK

**Keywords:** vascular endothelial growth factor, tumour marker, colorectal cancer

## Abstract

We aim to determine the clinical usefulness of pre-operative serum vascular endothelial growth factor (VEGF) as a predictor of outcome in patients undergoing curative resection for colorectal cancer. Serum VEGF was assayed by quantitative ELISA in 81 patients prior to curative resection for node-negative (n = 53) and node-positive (n = 28) disease. Median follow-up for patients without cancer death was 27 months (range 21–37). Pre-operative serum VEGF was significantly higher in patients who went on to develop metastases than those who did not (median, 713 pg ml–1 vs. 314 pg ml–1, P < 0.0001). Using multivariate Cox regression analysis, pre-operative serum VEGF was the most important prognostic factor independent of nodal status and adjuvant chemotherapy, and was superior to nodal status in predicting outcome (P < 0.00001). At 575 pg ml–1, pre-operative serum VEGF was 64% sensitive and 89% specific in predicting the development of metastases in curative resections, with a positive predictive value of 73% and a negative predictive value of 85%. Pre-operative serum VEGF is a powerful predictor of outcome following curative surgery for colorectal cancer. These data support the measurement of pre-operative serum VEGF as a method for selecting patients who require adjuvant therapy. © 2000 Cancer Research Campaign http://www.bjcancer.com


*British Journal of Cancer* (2000) **83**(11), 1425–1431
© 2000 Cancer Research Campaign
doi: 10.1054/ bjoc.2000.1508, available online at http://www.idealibrary.com on IDEAL®

http://www.bjcancer.com

### Pre-operative serum vascular endothelial growth factor can select patients for adjuvant treatment after curative resection in colorectal cancer

**K-F Chin, J Greenman, E Gardiner*, H Kumar, K Topping and J Monson**

Academic Surgical Unit and Department of Mathematics*, The University of Hull, Hull, UK

Summary We aim to determine the clinical usefulness of pre-operative serum vascular endothelial growth factor (VEGF) as a predictor of outcome in patients undergoing curative resection for colorectal cancer. Serum VEGF was assayed by quantitative ELISA in 81 patients prior to curative resection for node-negative (n = 53) and node-positive (n = 28) disease. Median follow-up for patients without cancer death was 27 months (range 21–37). Pre-operative serum VEGF was significantly higher in patients who went on to develop metastases than those who did not (median, 713 pg ml–1 vs. 314 pg ml–1, P < 0.0001). Using multivariate Cox regression analysis, pre-operative serum VEGF was the most important prognostic factor independent of nodal status and adjuvant chemotherapy, and was superior to nodal status in predicting outcome (P < 0.00001). At 575 pg ml–1, pre-operative serum VEGF was 64% sensitive and 89% specific in predicting the development of metastases in curative resections, with a positive predictive value of 73% and a negative predictive value of 85%. Pre-operative serum VEGF is a powerful predictor of outcome following curative surgery for colorectal cancer. These data support the measurement of pre-operative serum VEGF as a method for selecting patients who require adjuvant therapy. © 2000 Cancer Research Campaign http://www.bjcancer.com

**Keywords:** vascular endothelial growth factor; tumour marker; colorectal cancer

Although surgery offers the only reasonable expectation of cure for colorectal cancer, approximately 45% of all patients will go on to develop metastatic disease. The use of adjuvant chemotherapy (Moertel et al, 1995) and immunotherapy (Riethmuller et al, 1998) has been shown to provide survival benefit in node-positive disease. However, a considerable proportion of patients with node-negative disease will develop distant metastases (Olson et al, 1980) and adjuvant treatment is not routinely administered to this group of patients (Moertel et al, 1990). Although the pathological stage of disease, as defined by the Dukes' staging system and the depth of tumour infiltration (AJCC/UICC), can discriminate patients and yield information with prognostic value, its ability to predict the outcome of patients with intermediate stage of disease is poor. Patients with Dukes' stage B or C tumours that are prone, for whatever reason, to early disease recurrence would be likely to benefit from early identification and adjuvant therapy.

Tumour angiogenesis is essential for solid tumour growth, and facilitates tumour invasion and the dissemination of metastases (Folkman, 1990; Ellis and Fidler, 1996). Vascular endothelial growth factor (VEGF) is one of the most potent angiogenic proteins known. Our earlier work has shown that serum VEGF levels are raised in colorectal cancer patients ($n$ = 108) prior to surgical resections when compared with normal controls ($n$ = 136). ANOVA demonstrated a significant difference between the Dukes' stages ($P$ = 0.007), T stages ($P$ = 0.001) and UICC stages ($P$ = 0.001). In metastatic and node-positive disease, pre-operative serum VEGF was elevated compared with metastatic-free ($P$ = 0.03), node-negative disease ($P$ = 0.008) and controls ($P$ < 0.0005) (Kumar et al, 1998). In addition, in a series of 67 patients who had undergone curative resections for colorectal cancer, the VEGF concentrations fell post-surgery ($P$ < 0.0005) to levels comparable with the normal population. There was no fall in patients ($n$ = 15) who had undergone palliative resections ($P$ = 0.32) or in patients ($n$ = 12) who had undergone resections for benign disease ($P$ = 0.28) (Kumar et al, 1997). These data suggest that serum VEGF could be a potential tumour marker in colorectal cancer. The aim of this study was to assess the clinical usefulness of pre-operative serum VEGF as a predictor of outcome in patients who had undergone curative resections for colorectal cancer.

*Received 30 November 1999*
*Revised 3 August 2000*
*Accepted 15 August 2000*

*Correspondence to:* J Monson

### PATIENTS AND METHODS

### Study population

This is a prospective sequential cohort study in which 81 patients with primary colorectal cancer were examined. None of these patients had received blood transfusion, radiotherapy or chemotherapy prior to the study. All of these patients had undergone curative resection for colorectal cancer; 17 Dukes' A, 36 Dukes' B and 28 Dukes' C disease. All patients underwent standard radiological staging using either CT or MR abdominal and liver scanning. Scans were performed either pre-operatively or within 30 days of surgery in all cases. A single consultant pathologist staged all tumour samples according to TNM, UICC, JASS and Dukes' classification. 3 out of 36 patients with Dukes' B disease and 16 out of 28 patients with Dukes' C disease received adjuvant chemotherapy after surgery. The clinicians were unaware of the preoperative VEGF levels during the selection of patients for adjuvant chemotherapy, which was based on standard international guidelines. The local research ethics committee granted approval, and informed consent was obtained from all patients prior to their inclusion in this study.

### Storage of serum

7 ml of blood was collected pre-operatively. The serum was separated after 30 minutes of coagulation and immediately stored at –80˚C until immunoassay.

### VEGF assay

Serums were assayed for VEGF by quantitative sandwich ELISA (R&D Systems Europe, Oxford, UK). This assay will detect both soluble forms of VEGF (121 & 165). All of the serum samples and standards were assayed in duplicate. The minimum detectable level of VEGF was 9 pg $ml^{-1}$. In this study, 64 out of 81 patients also had their pre-operative carcino-embryonic antigen (CEA) levels determined.

### Follow-up programme after curative resection for colorectal cancer patients

All patients were included in a 5-year intensive radiological follow-up surveillance programme: rectal cancers were staged and followed-up with pelvic and liver MRI scans; colon cancer was staged and followed-up with liver CT scans. Our previous experience has shown no difference between CT and MRI scans in the detection of metastases and the protocol required patients to continue within a single imaging modality. The relevant scans were conducted at 3-monthly intervals for the initial 2 years and thereafter 6-monthly for the remaining 3 years. Patients also were subjected to routine surveillance colonoscopy or barium enema at yearly intervals. All patients complied with one of these two protocols, and only if they developed metastases, or died, were alternative courses of action taken.

### Statistical analysis

Analysis of differences in VEGF levels between the two groups of various prognostic factors was performed with Mann-Whitney U test. The optimal cut-off level of pre-operative serum VEGF was determined by an analytical method as described by Miller and Siegmund (1982). The influence of various clinico-pathological prognostic factors including the delivery of adjuvant chemotherapy on the time to development of metastases was assessed by univariate and multivariate analysis. Initially, univariate analysis was performed with Cox proportional hazard model to determine the factors related to the time to development of metastases. This was followed by forward stepwise multivariate Cox proportional hazard model to determine if a combination of prognostic factors provided a better estimate of the relative risk of time to development of metastases than any single variable. In the analysis of survival, curves for the disease-free survival and disease-specific survival were established by the Kaplan–Meier method and compared with the log-rank test. This also provides Kaplan–Meier product limit estimates of the survivor and cumulative hazard functions. The standard error estimates are based on Greenwood's formula. The confidence interval for survival uses an asymptotic maximum likelihood solution by transformation as recommended by Kalbfleisch and Prentice (1980). Cox's regression model was also used to examine the combinations of prognostic factors in the multivariate analysis of disease specific survival. All $P$ values are two-tailed.

### RESULTS

### Patient outcome

The median follow-up term for the patients without cancer death was 27 months (range 21–37). 25 patients developed distant metastases after curative resection for colorectal cancer. Among the 25 patients who developed distant metastases, 1 was staged Dukes' A, 11 Dukes' B and 13 Dukes' C. Peritoneal metastases occurred in 11 patients, and haematogeneous metastases occurred in 18 patients (13 liver metastases, 4 lung metastases and 1 bone metastases). 12 patients died from metastases. The cumulative metastases-free survival and cumulative disease-specific survival at 21 months of follow-up was 71% (95% CI 60–80; SE 5.2%) and 88% (95% CI 79–94; SE 3.7%), respectively.

### Association of the pre-operative serum VEGF with patient profile, and clinicopathological prognostic factors

No significant association was observed between pre-operative serum VEGF and age, gender, adjuvant chemotherapy, tumour site, tumour size, venous invasion or tumour differentiation. However, the level of serum VEGF was significantly associated with the following pathological staging; JASS staging, $P = 0.003$; UICC staging, $P = 0.0038$ and T stage, $P = 0.0036$, but not nodal status (Table 1).

### Association of the clinico-pathological prognostic factors with the time to metastases

The pre-operative serum VEGF levels were significantly higher in patients developing metastases than those who did not (713 pg $ml^{-1}$ vs. 314 pg $ml^{-1}$, $P < 0.0001$, 95% CI 189–438, Mann-Whitney U test). The prevalence of development of metastases increased as the serum VEGF increased with maximum prevalence at 600–800 pg $ml^{-1}$. The optimal cut-off level of pre-operative serum VEGF was determined at 575 pg $ml^{-1}$, for which the Chi-square value of the Cox's regression was maximal (Miller and Siegmund, 1982).

The specificity and positive predictive value of serum VEGF at 575 pg $ml^{-1}$ in predicting the development of metastases in comparison with the nodal status and CEA are shown in Table 2. Using the cut-off level of 575 pg $ml^{-1}$, serum VEGF could predict the development of metastases with positive predictive value of 73%, negative predictive value of 85%, sensitivity of 64% and specificity of 89%. Although CEA and nodal status do have some predictive capability, serum VEGF is by far the best predictor of outcome, specifically in terms of positive predictive value.

In comparison with the nodal status, patients with pre-operative serum VEGF levels higher than 575 pg $ml^{-1}$ have a much higher odds ratio and relative risk (Table 3). The odds of development of metastases for patients with pre-operative serum VEGF higher than 575 pg $ml^{-1}$ is 14.8 times that of patients with pre-operative serum VEGF levels lower than 575 pg $ml^{-1}$. On the other hand, the relative risks for the development of metastases for patients with VEGF higher than 575 pg $ml^{-1}$ is 4.8 when compared with those having VEGF lower than 575 pg $ml^{-1}$.

© 2000 Cancer Research Campaign

**Table 1** Association between preoperative serum VEGF and the clinico-pathological parameters

| Parameter | Patient No. | Preoperative serum VEGF (pg $ml^{-1}$) | |
|---|---|---|---|
| | | Median IQR | *P* value |
| Gender | | | |
| Male | 34 | 369 (224, 528) | NS |
| Female | 47 | 364 (193, 721) | |
| Age | | | |
| <65 | 30 | 369 (243, 568) | NS |
| ≥65 | 51 | 364 (192, 727) | |
| Adjuvant chemotherapy | | | |
| Yes | 19 | 371 (298, 540) | NS |
| No | 62 | 361 (214, 697) | |
| Tumour location | | | |
| Colon | 55 | 383 (291, 692) | NS |
| Rectum | 26 | 296 (168, 549) | |
| Tumour size | | | |
| <5 cm | 65 | 364 (207, 548) | NS |
| ≥5 cm | 16 | 547 (327, 758) | |
| Tumour differentiation | | | |
| Well, mucinous | 14 | 486 (371, 759) | NS |
| Moderately, poorly | 67 | 331 (198, 570) | |
| Vascular invasion | | | |
| Yes | 6 | 565 (291, 768) | NS |
| No | 75 | 364 (198, 631) | |
| JASS staging | | | |
| J1, J2 | 44 | 305 (170, 474) | 0.003 |
| J3, J4 | 37 | 522 (320, 746) | |
| Depth of infiltration | | | |
| T1, T2 | 21 | 291 (166, 367) | 0.0036 |
| T3, T4 | 60 | 425 (280, 734) | |
| UICC stages | | | |
| I | 19 | 250 (164, 364) | 0.0038 |
| II, III | 62 | 416 (290, 729) | |
| Lymph node metastases | | | |
| Negative | 53 | 331 (182, 590) | 0.07 |
| Positive | 28 | 438 (317, 716) | |
| Distant metastases | | | |
| Negative | 56 | 320 (182, 449) | <0.0001 |
| Positive | 25 | 713 (400, 795) | |
| Peritoneal metastases | | | |
| Negative | 70 | 338 (193, 542) | 0.0005 |
| Positive | 11 | 736 (524, 772) | |
| Haematogenous metastases | | | |
| Negative | 63 | 331 (192, 540) | 0.0033 |
| Positive | 18 | 635 (357, 830) | |
| Liver metastases | | | |
| Negative | 68 | 352 (204, 549) | 0.0219 |
| Positive | 13 | 590 (339, 877) | |

**Table 2** Validity of VEGF at 575 pg $ml^{-1}$ in predicting the development of metastases

| | Sensitivity (%) | Specificity (%) | PPV (%) | NPV (%) |
|---|---|---|---|---|
| CEA ≥ 5 ng $ml^{-1}$ | 48 | 63 | 38 | 71 |
| Node-positive | 52 | 73 | 46 | 77 |
| VEGF ≥ 575 pg $ml^{-1}$ | 64 | 89 | 73 | 85 |

PPV = Positive predictive value; NPV = Negative predictive value.

*© 2000 Cancer Research Campaign*

**Table 3** Prediction of development of metastases: VEGF *versus* nodal status

| | VEGF < 575 pg ml$^{-1}$ | VEGF ≥ 575 pg ml$^{-1}$ |
|---|---|---|
| M0 | 50 | 6 |
| M+ | 9 | 16 |

Odds ratio = 14.8, Relative risk = 4.8

| | Node-negative | Node-positive |
|---|---|---|
| M0 | 41 | 15 |
| M+ | 12 | 13 |

Odds ratio = 3, Relative risk = 2

| | Dukes' B, VEGF ≥ 575 pg ml$^{-1}$ | Dukes' B, VEGF < 575 pg ml$^{-1}$ |
|---|---|---|
| M0 | 20 | 5 |
| M+ | 5 | 6 |

Odds ratio = 4.8, Relative risk = 2.7.
M0 = Metastases free; M+ = Development of metastases.

Multivariate analysis of the time to metastases with Cox proportional hazard model was used to determine which clinicopathological prognostic variables provided an estimate of the relative risk for development of metastases. Using univariate analysis, serum VEGF and standard pathological prognostic factors (tumour size, venous invasion, JASS score, T classes, Dukes' stages and nodal status) all gave a significant estimate of relative risk of development of metastases (Table 4). In contrast, a single, pre-operative measurement of CEA (CEA ≥ 5 ng ml$^{-1}$) in 64 patients was not associated with the development of metastases. However, using forward stepwise multivariate Cox regression analysis, of all the variables included in the univariate analysis except serum CEA, serum VEGF was the most important prognostic factor in predicting the time to metastases ($P < 0.00001$; hazard ratio, 6.55; 95% CI, 2.73–15.68). The other independent prognostic factors were adjuvant chemotherapy ($P = 0.0382$; hazard ratio, 3.53; 95% CI, 1.07–11.65) and nodal status ($P = 0.0006$; hazard ratio, 4.65; 95% CI, 1.93–11.24): Table 5. In summary, the dichotomy of VEGF levels is more powerful in predicting the time to metastases than nodal status and will give additional predictive information to that available from nodal status and Dukes' staging (Figure 1). Although Dukes' staging is statistically significant in the univariate analysis, it is not a significant independent prognostic factor in the final model.

**Table 4** Univariate analysis of clinico-pathological factors in relation to time to development of metastases determined by Cox regression for patients undergoing curative resections for colorectal cancer

| Prognostic factors | Hazard ratio | 95% CI | *P* value |
|---|---|---|---|
| Age (≥65/<65) | 1.37 | 0.59–3.18 | 0.4601 |
| Gender (female/male) | 1.03 | 0.47–2.28 | 0.9349 |
| Size (≥5/<5) | 2.62 | 1.16–5.94 | 0.0209 |
| Differentiation (poor/well) | 0.59 | 0.23–1.47 | 0.2538 |
| Location (rectum/colon) | 0.40 | 0.14–1.16 | 0.0928 |
| Venous invasion (positive/negative) | 3.57 | 1.22–10.43 | 0.0200 |
| JASS stage (III, IV/I, II) | 2.96 | 1.28–6.87 | 0.0114 |
| Depth of infiltration (T III, IV/I, II) | 4.32 | 1.02–18.33 | 0.0472 |
| UICC stage (III, IV/I, II) | 3.65 | 0.86–15.48 | 0.0792 |
| Nodal status (positive/negative) | 2.52 | 1.15–5.53 | 0.0212 |
| Dukes' stages (overall) | | | 0.0478 |
| Dukes' C/Dukes'A | 9.58 | 1.25–73.25 | 0.0295 |
| Dukes' B/Dukes' A | 5.10 | 0.66–39.47 | 0.1190 |
| CEA (≥5 ng ml$^{-1}$ / <5 ng ml$^{-1}$): subset of 64 patients | 1.34 | 0.57–3.17 | 0.4996 |
| Adjuvant chemotherapy (no/yes) | 1.85 | 0.63–5.38 | 0.2619 |
| VEGF (≥575 pg ml$^{-1}$ / <575 pg ml$^{-1}$) | 7.14 | 3.11–16.38 | <0.00001 |

**Table 5** Multivariate analysis of various clinico-pathological factors in relation to time to development of metastases determined by forward stepwise Cox regression (final model) for patients undergoing curative resections for colorectal cancer

| Prognostic factors | Hazard ratio | 95% CI | *P* value |
|---|---|---|---|
| Adjuvant chemotherapy (no/yes) | 3.53 | 1.07–11.65 | 0.0382 |
| Nodal status (positive/negative) | 4.65 | 1.93–11.24 | 0.0006 |
| VEGF (≥575 pg ml$^{-1}$ / <575 pg ml$^{-1}$) | 6.55 | 2.73–15.68 | <0.00001 |

 *© 2000 Cancer Research Campaign*

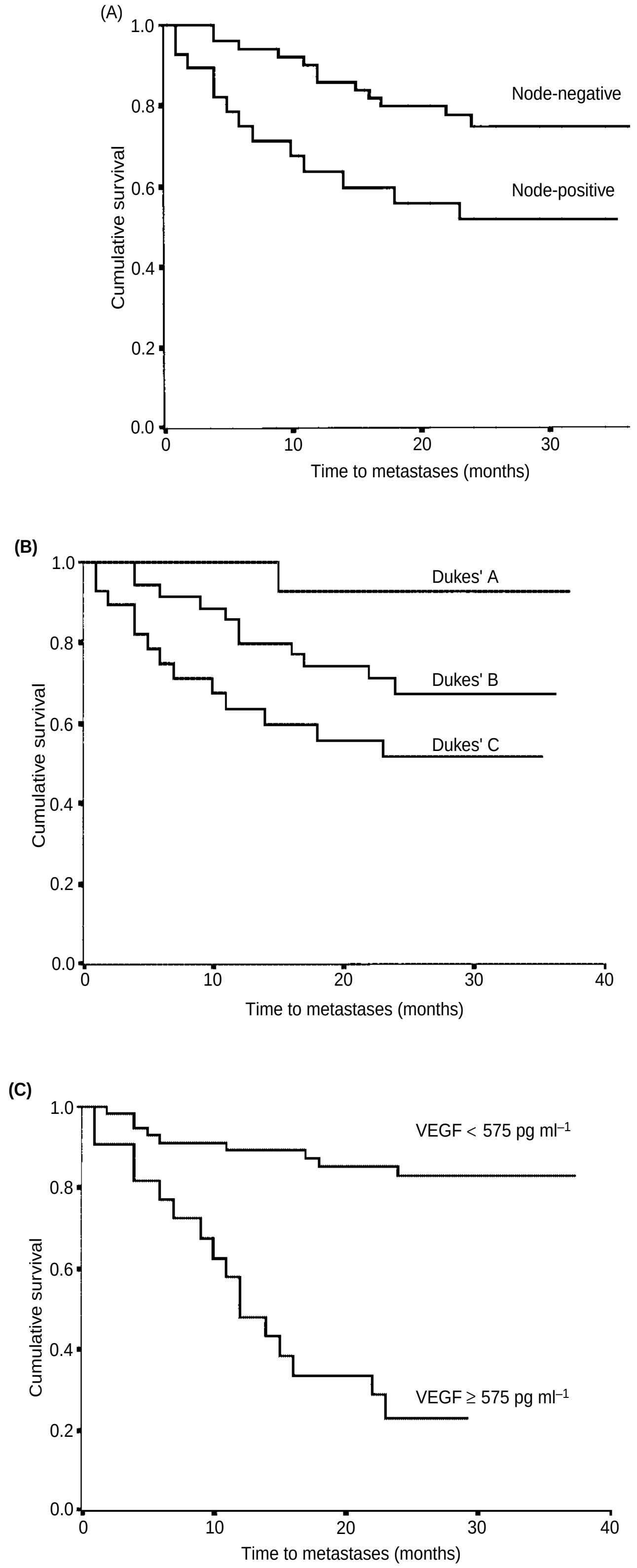

**Figure 1** Disease-free survival curves for colorectal cancer patients according to nodal status, Dukes' stage and pre-operative serum VEGF level. Kaplan Meier disease-free survival curves show that patients with: node-positive disease have a worse survival than those with node-negative disease (**A**); Log rank test, $P = 0.0164$; Dukes' stage C have a worse survival than Dukes' stage B (**B**); Log rank test $P = 0.0197$; pre-operative serum VEGF levels ≥575 pg $ml^{-1}$ have a worse survival than those patients with levels below 575 pg $ml^{-1}$ (**C**), Log rank test $P < 0.00001$.

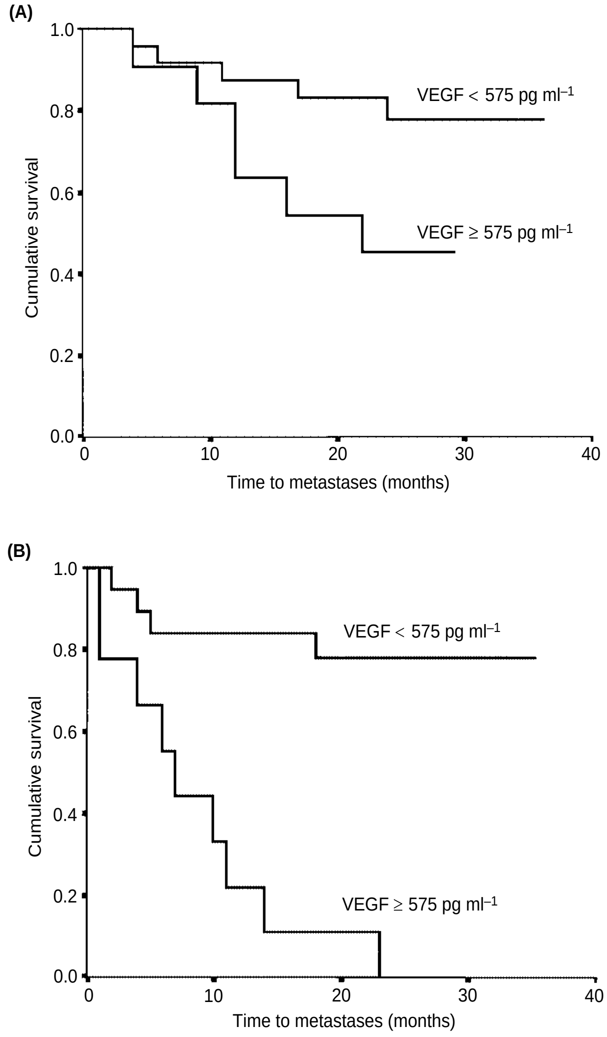

**Figure 2** Disease-free survival curves for patients with Dukes' B and Dukes' C disease according to VEGF levels. Kaplan Meier disease-free survival curves show that patients with Dukes' B or Dukes' C disease and pre-operative serum VEGF levels ≥575 pg $ml^{-1}$ have a worse survival (**A** and **B** respectively); Log rank test, $P < 0.00001$ in both cases.

Patients in this study were also subdivided into two groups according to their nodal status, and were investigated for any possible relationship between pre-operative serum VEGF and outcome. 12 of the 25 patients who developed metastases were staged as node-negative disease. 6 of these 12 patients had their pre-operative serum VEGF levels above 575 pg $ml^{-1}$ and therefore had a relative risk of at least 68% for developing metastases. None of these patients received adjuvant chemotherapy because of their nodal status as determined by pathological examination. In the Dukes' B disease group, patients with pre-operative serum VEGF higher than 575 pg $ml^{-1}$ have a much greater odds ratio and relative risk of development of metastases (odds ratio = 4.8, relative risk = 2.7) than the patients with node-positive disease alone. Within the node-negative group, the disease-free survival is greater in patients with VEGF levels lower than 575 pg $ml^{-1}$, than those with VEGF higher than 575 pg $ml^{-1}$ ($P = 0.0013$, log-rank test). Similarly, measurement of VEGF levels can further split the disease-free survival curve of Dukes' B patients, and provides additional information in predicting the development of metastases (Figure 2A, $P < 0.00001$, log-rank test).

In the node-positive group, all the patients who did not go on to develop metastases had pre-operative VEGF levels below 575 pg $ml^{-1}$. On the other hand, 9 out of 13 patients (69%) with node-positive disease who went on to develop metastases had their VEGF levels above 575 pg $ml^{-1}$. Thus, VEGF at 575 pg $ml^{-1}$ can correctly predict the disease-free survival within the node-positive disease group (Figure 2B, $P < 0.00001$, log-rank test). Furthermore, patients with serum VEGF higher than 575 pg $ml^{-1}$ were shown to have a poorer disease-specific survival rate ($P < 0.00001$, log-rank test).

Patients with Dukes' C have a poorer disease-specific survival than those patients with Dukes' B, while none of the patients with Dukes' A died from cancer ($P = 0.0044$, log-rank test). In addition, using the cut-off level of 575 pg $ml^{-1}$, pre-operative serum VEGF was also the most important independent prognostic factor in predicting the disease-specific survival using multivariate forward stepwise Cox regression analysis in this data set (VEGF, $P = 0.0004$; hazard ratio = 19.42; 95% CI 3.76–100.33, nodal status, $P = 0.0445$; hazard ratio = 4.41; 95% CI 1.04–18.72, tumour size, $P = 0.0460$; hazard ratio = 3.78; 95% CI 1.02–13.98).

### DISCUSSION

Our studies showed a significant correlation between the pre-operative level of serum VEGF and the probability of survival and of recurrence-free survival in colorectal cancer patients after curative resection. Patients with distant metastases at the time of surgery were excluded so that the evaluation of serum VEGF as a prognostic marker would be more specific and more appropriate for assessment of the clinical usefulness of this potential tumour marker. Previous studies have shown that VEGF expression is associated with the stage of progression and metastases in colorectal cancer and may play a predictive role in the prognosis of the disease (Takahashi et al, 1997; Ishigami et al, 1998). Salven and colleagues have shown that high pre-treatment serum VEGF predicts poor response to chemotherapy and poor survival in small-cell lung cancer (Salven et al, 1998). A similar study in patients with non-Hodgkin's lymphoma also demonstrated that patients with higher than median serum VEGF pre-treatment have a poorer survival rate than those patients with serum levels below the median value (Salven et al, 1997). Our current study on colorectal cancer patients undergoing curative resection has demonstrated that pre-operative serum VEGF is the most important independent prognostic factor for determining the future development of metastases, disease-free survival and disease-specific survival. The higher the level of serum VEGF prior to curative resection, the greater the risk of developing distant metastases in the future.

Our studies have indicated that angiogenesis plays a vital role in the development of metastases (Ellis and Fidler, 1996; Folkman and Shing, 1992). The higher levels of serum VEGF in those patients that went on to develop metastases, despite curative resection, might reflect the increased tumour load and hence an increased metastatic potential for purely stochastic reasons. In addition, it has been shown that increased serum VEGF levels were significantly correlated with high microvessel density and VEGF expression in primary breast tumour tissue (Yamamoto et al, 1996). Therefore, it is likely that the high levels of serum VEGF reflect the degree of angiogenesis at the tumour site, which facilitates the dissemination of cancer cells into the circulation (Folkman, 1994). It is also probable that the relatively high level of serum VEGF systemically may provide the required environment for establishment of adequate angiogenesis at distant metastatic sites. The significant association between the pre-operative VEGF levels and the development of haematogenous and peritoneal metastases reported here supports this hypothesis. On the contrary, the lack of association between the pre-operative serum VEGF levels and the lymph node metastases suggests that VEGF may not facilitate metastatic spread via the lymphatic vessels. However, Yonemura et al (1999) have found a strong correlation between VEGF-C tissue expression status and the grade of lymph node metastases in gastric cancer. This finding suggests that VEGF-C rather than VEGF may be important in the development of lymphatic spread.

Of all the patients presented with colorectal cancer, approximately 70% are resectable with curative intent, and 45% are indeed cured by primary resection. However, the cancer in the remaining 25% of curative resections recurs, most commonly during the first two years after resection (August et al, 1984). The majority of mortality from colorectal cancer is attributed to the development of metastases. The prognosis of metastatic cancer is influenced by a variety of clinico-pathological features present at the initial diagnosis (Williams et al, 1988; Deans et al, 1992; Hermanek et al, 1995). The most widely accepted prognostic factor in colorectal cancer is the presence of lymph node metastases (Cohen et al, 1991), which is pivotal for the decisions regarding adjuvant chemotherapy. However, a considerable proportion of patients with node-negative disease goes on to develop metastases, 23% in this study. Recently, IMPACT B2 investigators have concluded that there is no good evidence to support the routine use of adjuvant fluorouracil and folinic acid in B2 (T3 and T4, N0 M0) colon cancer. Instead, they have indicated the need for more pertinent biological markers to identify a subgroup of patients at high risk of disease recurrence (IMPACT B2 investigators, 1999). The data from our study has shown that pre-operative serum VEGF levels can provide additional information to Dukes' staging in the prediction of a sub-group of patients who are most likely to develop aggressive metastases during the first 2 years following surgical resection; the period of time when patients are most vulnerable to disease recurrence (Pestana et al, 1964; Polk and Spratt, 1971; August et al, 1984). This study indicates that patients with high pre-operative serum VEGF levels were exposed to high risk of early aggressive metastases. Pre-operative selection of this sub-group of patients for neo-adjuvant or immediate delivery of aggressive adjuvant chemotherapy after surgery might prolong the survival of these patients. In addition, pre-operative identification of patients with Dukes' B tumours with a high risk of metastases by a single measurement of serum VEGF is highly likely to improve the survival of this group of colorectal cancer patients by the use of adjuvant therapy following surgery. Furthermore, identification of such patients can facilitate the surgical decision regarding local versus radical resection and neoadjuvant radio-chemotherapy, in patients with rectal cancer. On the other hand, pre-operative serum VEGF is also useful for selecting patients with node-positive disease that would not benefit from aggressive adjuvant treatments after surgery. These data suggest that serum VEGF can stage Dukes' B and C disease further, and may become the tumour marker of choice to select patients for adjuvant therapy.

© 2000 Cancer Research Campaign

### ACKNOWLEDGEMENTS

Kin-Fah Chin is supported by the Frances and Augustus Newman Foundation and the Albert McMasters award from British Medical Association.

### REFERENCES

August DA, Ottow RT and Sugarbaker PH (1984) Clinical perspective of human colorectal cancer metastasis. *Cancer Metastasis Rev* **3**(4): 303–324

Cohen AM, Tremiterra S, Candela F, Thaler HT and Sigurdson ER (1991) Prognosis of node-positive colon cancer. *Cancer* **67**(7): 1859–1861

Deans GT, Parks TG, Rowlands BJ and Spence RA (1992) Prognostic factors in colorectal cancer. *Br J Surg* **79**(7): 608–613

Ellis LM and Fidler IJ (1996) Angiogenesis and metastasis. *Eur J Cancer* **32A**(14): 2451–2460

Folkman J (1990) What is the evidence that tumours are angiogenesis dependent? *J Natl Cancer Inst* **82**(1): 4–6

Folkman J (1994) Angiogenesis and breast cancer. *J Clin Oncol* **12**(3): 441–443

Folkman J and Shing Y (1992) Angiogenesis. *J Biol Chem* **267**(16), 10931–10934

Hermanek P, Wiebelt H, Staimmer D and Riedl S (1995) Prognostic factors of rectum carcinoma – experience of the German multicentre study of SGCRC. German Study Group Colo-Rectal Carcinoma. *Tumori* **81**(3 Suppl): 60–64

IMPACT B2, the International Multicentre Pooled Analysis of B2 Colon Cancer Trials Investigators (1999) Efficacy of adjuvant fluorouracil and folinic acid in B2 colon cancer. *J Clin Oncol* **17**: 1356–1363

Ishigami SI, Arii S, Furutani M, Niwano M, Harada T, Mizumoto M, Mori A, Onodera H and Imamura M (1998) Predictive value of vascular endothelial growth factor (VEGF) in metastases and prognosis of human colorectal cancer. *Br J Cancer* **78**(10): 1379–1384

Kalbfleisch JD and Prentice RL (1980) *Statistical Analysis of Failure Time Data*, New York: Wiley.

Kumar H, Heer K, Lee PW, Duthie GS, MacDonald AW, Greenman J, Kerin MJ and Monson JR (1997) Utilisation of serum VEGF to predict curative colorectal cancer resections. *GUT* **39**: 964 (suppl 3)

Kumar H, Heer K, Lee PW, Duthie GS, MacDonald AW, Greenman J, Kerin MJ and Monson JR (1998) Preoperative serum vascular endothelial growth factor can predict stage in colorectal cancer. *Clin Cancer Res* **4**(5): 1279–1285

Miller R and Siegmund D (1982) Maximally selected chi square statistics. *Biometrics* **38**: 1011–1016

Moertel CG, Fleming TR, Macdonald JS, Haller DG, Laurie JA, Goodman PJ, Ungerleider JS, Emerson WA, Tormey DC, Glick JH, Veeder MH and Mailliard JA (1990) Levamisole and fluorouracil for adjuvant therapy of resected colon carcinoma. *N Engl J Med* **322**(6): 352–358

Moertel CG, Fleming TR, Macdonald JS, Haller DG, Laurie JA, Goodman PJ, Ungerleider JS, Emerson WA, Tormey DC, Glick JH, Veeder MH and Mailliard JA (1995) Fluorouracil plus levamisole as effective adjuvant therapy after resection of stage III colon carcinoma: A final report. *Ann Intern Med*; **122**(5): 321–326

Olson RM, Perencevich NP, Malcolm AW, Chaffey JT and Wilson RE (1980) Patterns of recurrence following curative resection of adenocarcinoma of the colon and rectum. *Cancer* **45**(12): 2969–2974

Pestana C, Reitemeier RJ, Moertel CG, Judd ES and Dockerty MB (1964) The natural history of carcinoma of the colon and rectum. *Am J Surg* **108:** 826–829

Polk HC Jr and Spratt JS Jr. (1971) Recurrent colorectal carcinoma: detection, treatment, and other consideration. *Surgery* **69**: 9–23

Riethmuller G, Holz E, Schlimok G, Schmiegel W, Raab R, Hoffken K, Gruber R, Funke I, Pichlmaier H, Hirche H, Buggisch P, Witte J and Pichlmayr R (1998) Monoclonal antibody therapy for resected Dukes' C colorectal cancer: seven-year outcome of a multicenter randomized trial. *J Clin Oncol* **16**(5): 1788–1794

Salven P, Teerenhovi L and Joensuu H (1997) A high pretreatment serum vascular endothelial growth factor concentration is associated with poor outcome in non-Hodgkin's lymphoma. *Blood* **90**(8): 3167–3172

Salven P, Ruotsalainen T, Mattson K and Joensuu H (1998) High pretreatment serum level of vascular endothelial growth factor (VEGF) is associated with poor outcome in small cell lung cancer. *Int J Cancer* **79**(2): 144–146

Takahashi Y, Tucker SL, Kitadai Y, Koura AN, Bucana CD, Cleary KR and Ellis LM (1997) Vessel counts and expression of vascular endothelial growth factor as prognostic factors in node-negative colon cancer. *Arch Surg* **132**(5): 541–546

Williams NS, Jass JR, and Hardcastle JD (1988) Clinicopathological assessment and staging of colorectal cancer. *Br J Surgery* **75**(7): 649–652

Yamamoto Y, Toi M, Kondo S, Matsumoto T, Suzuki H, Kitamura M, Tsuruta K, Taniguchi T, Okamoto A, Mori T, Yoshida M, Ikeda T and Tominaga T (1996) Concentrations of vascular endothelial growth factor in sera of normal control and cancer patients. *Clin Cancer Res* **2**(5), 821–826

Yonemura Y, Endo Y, Fujita H, Fushida S, Ninomiya I, Bandou E, Taniguchi K, Miwa K, Ohoyama S, Sugiyama K and Sasaki T (1999) Role of vascular endothelial growth factor C expression in the development of lymph node metastasis in gastric cancer. *Clin Cancer Res* **5**(7), 1823–1829

© 2000 Cancer Research Campaign 
